# Condition-Linked Opioid Prescribed-Medicine Events and Subsequent Emergency Department Use Among Adults With Surgical or Procedural Events: A Longitudinal Medical Expenditure Panel Survey Study

**DOI:** 10.7759/cureus.116608

**Published:** 2026-09-20

**Authors:** Dhruv Patel, Akshita Wadhwa, Karina V Eolyan, Areesh Lalani, Azim Lalani

**Affiliations:** 1 Osteopathic Medicine, Nova Southeastern University Dr. Kiran C. Patel College of Osteopathic Medicine, Clearwater, USA; 2 Family Medicine, Southwest Florida Medicine, Bradenton, USA

**Keywords:** effect modification, emergency department use, longitudinal survey, opioid prescriptions, poverty, surgery-associated conditions

## Abstract

Background

We examined whether an observed opioid prescribed-medicine event linked to the same condition as a surgical/procedural event in year 1 was associated with all-cause emergency department (ED) use in year 2 and whether this association differed by poverty status. These medication events represent medicines reported as purchased or otherwise obtained; the linkage does not establish postoperative timing.

Materials and methods

We pooled four two-year Medical Expenditure Panel Survey (MEPS) panels: 2017-2018, 2018-2019, 2020-2021, and 2021-2022. Adults were eligible when a year-1 surgical/procedural event was linked to a condition record. The exposure was one or more observed year-1 opioid prescribed-medicine events linked to that same condition, compared to none. The primary outcome was the year-2 annual all-cause ED visit count, and the secondary outcome was any year-2 ED use. Survey-weighted quasi-Poisson and logistic models were adjusted for demographic characteristics, poverty, insurance, year-1 utilization, health status, surgical settings, surgical-event burden, and panel. Adjusted outcomes were standardized over the analytic population. A prespecified interaction compared people below with at or above 200% of the federal poverty level (FPL).

Results

Among 4,468 eligible adults, 4,454 (99.7%) were complete cases, and 816 of 4,454 (18.3%) had an observed condition-linked opioid prescribed-medicine event. Adjusted mean annual ED visits were 0.336 (95% confidence interval {CI}: 0.275-0.412) with and 0.345 (95% CI: 0.311-0.382) without a linked opioid prescribed-medicine event. The adjusted mean ratio was 0.975 (95% CI, 0.784-1.211; p=0.8165), and the mean difference was -0.009 visits (95% CI, -0.082 to 0.065; p=0.8150). Any year-2 ED use occurred in 194 of 816 exposed participants (survey-weighted, 22.5%; 95% CI, 18.9%-26.1%) and 845 of 3,638 comparators (survey-weighted, 21.5%; 95% CI, 19.7%-23.3%). The exposure-by-poverty interaction did not provide evidence of heterogeneity (ratio of ratios, 1.112; 95% CI, 0.751-1.649; F=0.284; p=0.5943). The adjusted risk difference for any ED use was -1.9 percentage points (95% CI, -5.7 to 1.8; p=0.3073).

Conclusions

No clear adjusted association was detected between an observed year-1 condition-linked opioid prescribed-medicine event and year-2 annual all-cause ED use. The estimates were imprecise and do not establish equivalence, the absence of an association, or socioeconomic equity. The exposure cannot be classified as postoperative because the procedure-medication sequence was not established.

## Introduction

Current US guidance on opioid prescribing for pain sets no uniform dose or duration rule, emphasizing individualized treatment, shared decision-making, and periodic reassessment instead [1]. In surgical care, that leaves the balance between adequate analgesia and the potential harms of opioid therapy to be struck case by case. Multimodal pain management can combine opioid and nonopioid medications with regional or other techniques, but identifying this strategy requires component-level clinical information that is not available in most household surveys [2,3]. Consequently, an observed prescribed-medicine event should not be interpreted as a complete description of the care pathway in which the medicine was obtained.

Medical Expenditure Panel Survey (MEPS) studies have described opioid and nonpharmacologic treatment patterns among adults with chronic or surgical pain [4]. Investigators have also used broad inpatient, outpatient, office-based, and emergency department (ED) procedure indicators to identify opioid prescriptions associated with surgery in adolescents [5]. These studies illustrate the use of MEPS for analyses of broad surgical/procedural indicators and prescribed-medicine records. However, the same-year linkage used here does not establish the sequence of individual medication acquisitions and procedures or define a postoperative window.

Prior MEPS analyses have examined opioid prescriptions in relation to annual ED or hospital-based service use [6,7]. Longitudinal panel studies have also ordered health status or opioid exposure in one year and outcomes in the next [8,9]. This ordering can reduce ambiguity between the exposure year and the subsequent outcome year, but it does not remove confounding by indication, especially when clinical severity and prescribing are measured during the same year. Socioeconomic context adds another concern. Previous research has reported differences in opioid prescribing across race, income, insurance, and clinical settings [10-12]. A recent longitudinal MEPS study also examined variation in surgery-associated financial outcomes by payer and income [13]. Whether the association between an opioid prescribed-medicine event linked to a surgical/procedural condition and later acute care use differs by poverty status remains uncertain.

Claim-based studies answer a narrower postoperative question by anchoring patients to exact procedures, dispensing dates, baseline opioid status, and fixed follow-up windows [14-16]. MEPS instead offers a complementary national perspective that combines conditions, health status, utilization, and socioeconomic measures across two-year panels. The present analysis combines same-year condition-event-medication linkage, a subsequent-year annual ED outcome, and a prespecified poverty interaction.

The primary objective was to estimate the association between one or more observed year-1 opioid prescribed-medicine events linked to the same condition as a year-1 surgical/procedural event and the year-2 annual all-cause ED visit count among eligible US adults. Secondary objectives were to assess any year-2 ED use and to test whether the count association differed between people below and at or above 200% of the federal poverty level (FPL). The study estimated longitudinal associations; the same-year condition linkage did not establish that medication acquisition followed the procedure.

## Materials and methods

Study design and data sources

We conducted a repeated-panel longitudinal observational cohort analysis using the Agency for Healthcare Research and Quality MEPS Household Component public-use files [17]. MEPS represents the US civilian noninstitutionalized population and follows overlapping panels through five interview rounds over two calendar years. We pooled four independent two-year panels: Panel 22 (2017 exposure year and 2018 outcome year), Panel 23 (2018 and 2019), Panel 25 (2020 and 2021), and Panel 26 (2021 and 2022). The prespecified analysis included Panels 22, 23, 25, and 26. Panel 24 (2019-2020) was not included, although a two-year longitudinal file is available as HC-225; inference is therefore restricted to the four selected panel periods. Panel 25 was retained in the primary analysis, and its exclusion was prespecified as a sensitivity analysis because its exposure year overlapped the first year of the coronavirus disease 2019 pandemic.

For each panel, we linked the longitudinal file to year-1 inpatient, emergency-room, outpatient, office-based, prescribed-medicine, event-condition-link, and condition public-use files. Common variance estimation variables were obtained from the HC-036 pooled variance file [18]. The exact public-use file crosswalk is provided in the Appendices.

The protocol and reporting map were frozen before any adjusted comparative estimate was reviewed; earlier runs failed for environment and implementation reasons without producing comparative estimates. Reporting was guided by the Strengthening the Reporting of Observational Studies in Epidemiology statement, with relevant linkage and coding-algorithm concepts from the REporting of studies Conducted using Observational Routinely-collected health Data statement [19,20]. All post-protocol implementation decisions are documented in the supplement and did not change the approved population, exposure, comparator, primary outcome, estimands, or interpretation boundary.

Study population and cohort construction

The source universe included target-panel members with a positive longitudinal weight. Adults were eligible when they completed all five rounds, were aged 18 years or older in year 1, and had at least one year-1 event carrying a surgical or procedural indicator. A qualifying event had "ANYOPER=1" in the inpatient file or "SURGPROC=1" in the emergency-room, outpatient, or office-based file. The event also had to link to a condition record through the person, event, and condition identifiers.

These indicators cover a broad range of surgical/procedural encounters across inpatient, emergency-room, outpatient, and office-based settings. Eligibility did not require a particular operation or establish comparable procedure complexity across participants.

We included every record satisfying these criteria rather than choosing a sample size from an anticipated effect. Before modeling, we required more than 100 residual survey-design degrees of freedom, at least 50 observations and 30 ED-positive observations per categorical adjustment level after clinically defensible collapsing, and at least 10 ED-positive participants per fitted parameter as a conservative diagnostic. Figure 1 shows aggregate cohort construction, with panel-specific counts in the Appendices.

**Figure 1:**
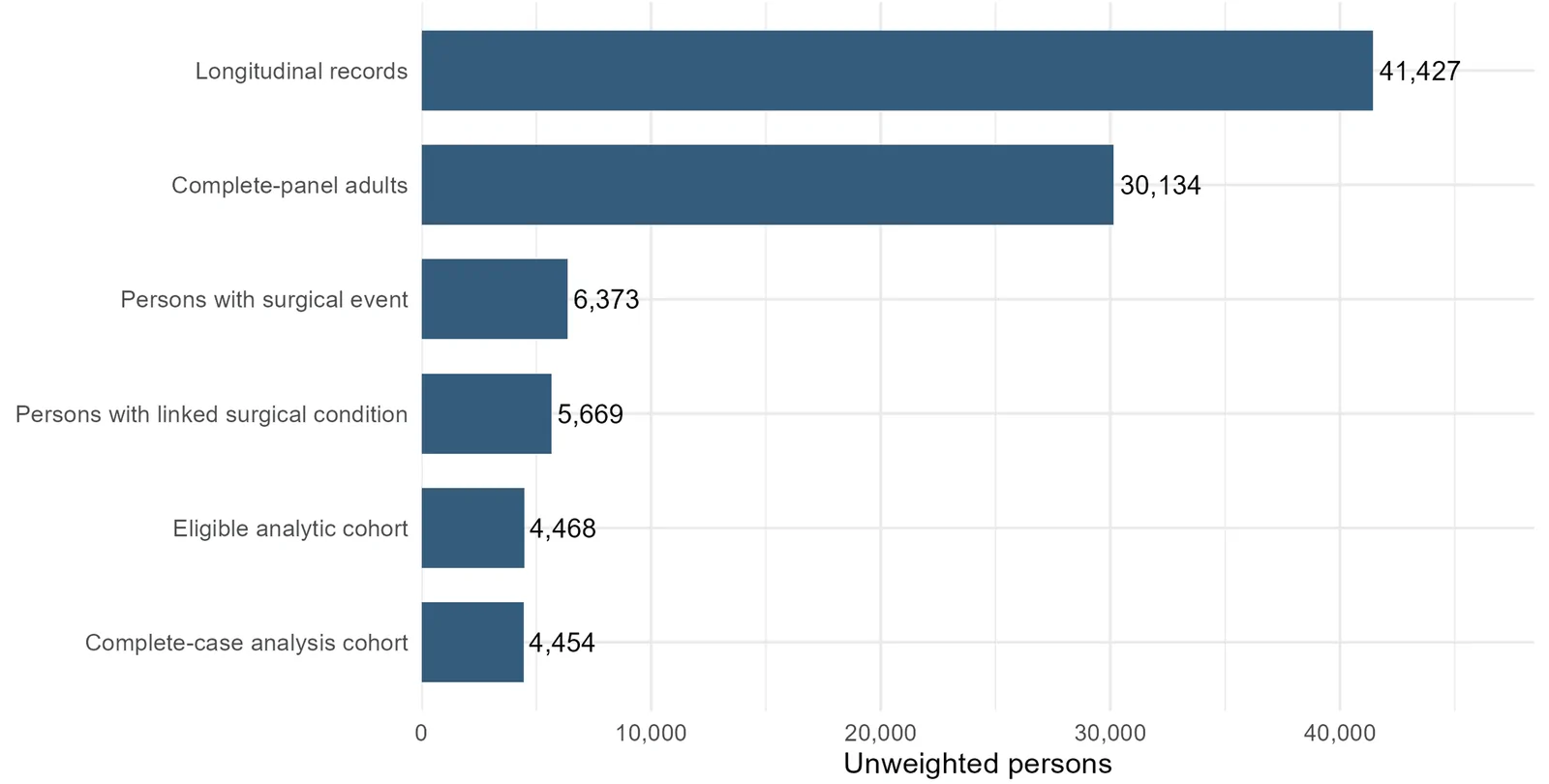
Aggregate cohort construction across four longitudinal Medical Expenditure Panel Survey panels Counts are unweighted persons, summed across Panels 22, 23, 25, and 26. Surgical-event and linked-condition counts were obtained from the year-1 event and condition files before intersection with complete-panel adults having positive longitudinal weights; they are not successive exclusions from the 30,134 complete-panel adults. That intersection yielded 4,468 eligible adults, of whom 4,454 were complete cases. "Surgical event" in the figure includes the broad surgical/procedural indicators defined in this section. Panel-specific counts appear in the Appendices

Surgical/procedure event, condition, and prescription linkage

Within each panel, qualifying surgical/procedural events were linked to the event-condition file using DUPERSID and EVNTIDX. Distinct DUPERSID-CONDIDX pairs were retained only when they also existed in the year-1 condition file. Prescribed-medicine links with EVENTYPE=8 were matched to these condition pairs, and their EVNTIDX values were matched to LINKIDX in the prescribed-medicines file for the same DUPERSID. All available Multum TC hierarchy fields were searched; a record was classified as opioid when any field contained class 60 or 191. Participants were exposed if at least one qualifying opioid record was linked to any qualifying condition. Multiple conditions, events, or medicine acquisitions did not create additional participants or change the binary any-versus-none definition.

Prescribed-medicine events represent medicines reported by households as purchased or otherwise obtained, rather than a count of prescriptions written by clinicians or confirmed medication consumption [17]. The linkage establishes a shared condition within year 1, but it does not establish whether medication acquisition preceded or followed the surgical/procedural event or was specifically for postoperative pain. MEPS includes coarse timing information, such as the acquisition interview round and the month and year a medicine was first used; these fields were not used to order acquisitions and procedures in this analysis, and first-use dates are not acquisition dates for subsequent refills. The comparator included people with no observed qualifying linked opioid event, including people with linked nonopioid medicines, medicines linked to other conditions, or no qualifying linked medicine.

Exposure, outcomes, and socioeconomic modifier

The primary exposure was binary: one or more observed condition-linked opioid prescribed-medicine events in year 1 versus none. This indicator did not incorporate dose, duration, acquisition count, specific opioid, strength, morphine milligram equivalents, or timing relative to a procedure. Four treatment-pattern categories (opioid only, nonopioid only, both, and neither) were retained for descriptive feasibility work but were not used for adjusted comparisons because sparse cells failed the prespecified precision thresholds.

The primary outcome was the year-2 annual all-cause ED visit count ("ERTOTY2"), selected to measure subsequent overall acute-care utilization during a calendar year following the exposure year. The secondary outcome was any year-2 ED use, defined as one or more visits. This year-separated outcome does not measure a fixed interval after an individual procedure or medicine acquisition. The annual total does not establish why an ED encounter occurred and was not interpreted as a postoperative revisit, pain-related encounter, opioid-related adverse event, or procedural complication.

The prespecified socioeconomic modifier was year-1 family income relative to the FPL. MEPS poverty categories 1-3 were combined as below 200% FPL and categories 4-5 as at or above 200% FPL. A three-level specification (below 200%, 200%-399%, and at or above 400% FPL) was examined in sensitivity analysis.

Covariates

Covariates were selected a priori from demographic, health, utilization, and surgical-event domains used in related MEPS studies. The main models included age as a natural spline with three degrees of freedom; sex; race and ethnicity; census region; two-level poverty; insurance; year-1 ED visits; inpatient discharges; outpatient visits; office-based visits; self-rated physical and mental health; walking limitation; cancer; indicators for inpatient, emergency-room, outpatient, and office-based surgical settings; the number of unique year-1 surgical/procedural events; and panel.

Year-1 utilization counts and surgical-event burden were transformed as "log(1+x)." For adjustment, non-Hispanic Asian and non-Hispanic other/multiple-race categories were combined after the separate Asian category had only six ED-positive participants. Public-only and uninsured groups were combined as no private coverage after the uninsured category had 17 ED-positive participants. Table 1 retains the released five-category race/ethnicity and three-category insurance descriptions. Race and ethnicity were treated as social measures rather than biological attributes.

**Table 1:** Survey-weighted characteristics of the complete-case cohort In the "overall" column, N is the exact unweighted number of participants in the complete-case cohort (N=4,454), and the value in parentheses is the corresponding survey-weighted percentage; rows reporting a mean give the survey-weighted mean, followed by the unweighted N contributing to it. Because the Medical Expenditure Panel Survey applies complex survey weights, N divided by 4,454 will not equal the weighted percentage. The exposure-group columns report survey-weighted percentages or means for the unweighted group sizes shown in the column headings (n=3,638 and n=816). Within each categorical block, the overall N values sum to 4,454. The mutually exclusive highest-acuity setting is descriptive; the adjusted models used four concurrent setting indicators. Released race/ethnicity and insurance categories are shown for description. Adjustment combined non-Hispanic Asian with non-Hispanic other/multiple race and combined public-only with uninsured coverage to satisfy prespecified model-level event thresholds. Race and ethnicity were treated as social measures rather than biological attributes. "Linked opioid" denotes an observed year-1 opioid prescribed-medicine event linked to a condition also linked to a qualifying surgical/procedural event; "any" and "no" denote the presence or absence of at least one such event ED: emergency department

Characteristic	Overall, N (%) or mean (N=4,454)	No observed linked opioid (n=3,638)	Any observed linked opioid (n=816)	Standardized difference
Age, mean years	57.23 (N=4,454)	58.12	53.01	-0.295
Female sex	2,580 (56.9%)	56.4%	59.3%	0.059
Hispanic	464 (8.7%)	8.7%	9.0%	0.013
Non-Hispanic White	3,326 (78.4%)	78.5%	77.5%	-0.024
Non-Hispanic Black	454 (7.5%)	7.2%	8.9%	0.063
Non-Hispanic Asian	83 (2.7%)	3.0%	1.2%	-0.108
Non-Hispanic other/multiple race	127 (2.7%)	2.6%	3.3%	0.045
Northeast	794 (18.4%)	18.7%	16.9%	-0.046
Midwest	1,061 (24.0%)	23.0%	28.7%	0.134
South	1,546 (35.8%)	35.9%	35.4%	-0.009
West	1,053 (21.8%)	22.4%	18.9%	-0.084
Below 200% federal poverty level	1,305 (24.6%)	24.1%	27.0%	0.067
Any private insurance	2,776 (68.1%)	67.6%	70.1%	0.054
Public insurance only	1,608 (30.5%)	30.9%	28.5%	-0.052
Uninsured	70 (1.4%)	1.4%	1.3%	-0.008
Year-1 ED visits, mean	0.51 (N=4,454)	0.47	0.69	0.188
Year-1 inpatient discharges, mean	0.38 (N=4,454)	0.33	0.61	0.365
Year-1 outpatient visits, mean	2.51 (N=4,454)	2.24	3.82	0.277
Year-1 office-based visits, mean	15.57 (N=4,454)	14.97	18.44	0.204
Fair/poor physical health	1,042 (20.4%)	19.6%	24.1%	0.111
Fair/poor mental health	475 (9.3%)	9.2%	9.7%	0.017
Walking limitation	1,321 (25.9%)	23.9%	35.2%	0.257
Cancer diagnosis	1,226 (26.5%)	27.9%	19.8%	-0.185
Highest-acuity setting: inpatient	1,090 (23.2%)	18.9%	44.1%	0.597
Highest-acuity setting: emergency room	193 (4.1%)	4.4%	2.7%	-0.085
Highest-acuity setting: outpatient	1,242 (28.0%)	26.6%	34.7%	0.181
Highest-acuity setting: office-based	1,929 (44.7%)	50.1%	18.5%	-0.636
Year-1 surgical events, mean	2.09 (N=4,454)	2.06	2.24	0.078

The exposure accumulated throughout year 1. Annual utilization, surgical/procedural setting, and event burden were measured during the same year and could precede or follow medication acquisition. Health-status measures were recorded in round 1, but their temporal position relative to all year-1 medication acquisitions was not established. These variables may reflect preexisting risk, contemporaneous severity, or downstream consequences of care. Adjustment therefore defines an associational contrast and does not identify a causal effect.

Survey design and statistical analysis

We constructed the survey design on the full positive-weight pooled longitudinal universe and then analyzed the eligible cohort as a survey domain. Strata and primary sampling units were "STRA9623" and "PSU9623" from HC-036. The selected panels span two longitudinal variance structures: Panels 22 and 23 precede the change introduced with Panel 24, whereas Panels 25 and 26 use the later structure. We therefore used the common HC-036 pooled variance variables for this combination [18]. The analysis weight was each panel's longitudinal weight divided by four, so weighted estimates represent an average across the four included panel periods. An explicit adjusted policy for lonely sampling units was set but was not invoked because no lonely strata were present.

The primary model was a survey-weighted quasi-Poisson log-mean regression for the year-2 ED count. The prespecified log-mean model was fitted with survey weights and design-based robust variance, allowing inference without requiring the equality of the count mean and variance. Negative-binomial models also estimate a conditional mean but use a different mean-variance structure; targeting the mean alone does not rule them out. The quasi-Poisson model was retained because the prespecified convergence, sparsity, influence, and calibration checks did not trigger a change in specification. A high observed zero proportion alone was not treated as evidence of a distinct zero-inflation process. These diagnostics support retaining the prespecified model but do not establish its superiority to every alternative. The primary adjusted contrast was the standardized mean count ratio for any versus no observed condition-linked opioid prescription. The corresponding standardized mean difference was reported as an absolute estimand.

The prespecified effect-modification model added the exposure-by-poverty interaction. We used the one-degree-of-freedom interaction Wald test as the inferential test of heterogeneity and calculated poverty-specific standardized means, ratios, and differences. We did not infer a difference by comparing separate subgroup p-values. A survey-weighted quasibinomial logistic model estimated the secondary any-ED outcome.

For standardization, we predicted each complete-case participant's outcome under each exposure value while retaining all other observed covariates and then averaged those predictions with the survey weights. Poverty-specific estimates were standardized within the corresponding poverty domain. Taylor linearization combined the coefficient influence functions with the survey-weighted empirical distribution of covariates; this propagated uncertainty from both model estimation and the standardization population into means, ratios, differences, and 95% confidence intervals (CIs). Methods followed the design-based framework implemented in the R "survey" package [21].

We report two-sided p-values and 95% CIs. The primary count association, one prespecified poverty interaction, and secondary binary outcome formed the inferential hierarchy. Sensitivity and exploratory analyses were interpreted as robustness checks rather than separate confirmatory tests.

Data extraction and linkage used Python 3.10.9 (Python Software Foundation, Fredericksburg, VA). Survey analyses used R 4.6.1 with the survey package version 4.5. The exact programs, variable dictionary, protocol, and recorded environment are available through the code and material statement in the Appendices.

Missing data, diagnostics, and sensitivity analyses

Complete-case analysis was permitted when cumulative loss was no more than 5%, no main covariate had more than 2% missingness, and the absolute difference in missingness by exposure was no more than five percentage points. Otherwise, comparative modeling would have stopped pending an approved survey-compatible imputation plan.

Required diagnostics included convergence and finite coefficients, residual design degrees of freedom, quasi-Poisson dispersion, observed zero proportion, sparse levels, ED-positive participants per parameter, the model-matrix condition number, sampling-weight influence, and observed-versus-predicted calibration overall, by exposure, and across weighted prediction groups. Descriptive estimates were not reportable when an unweighted cell contained fewer than 30 observations or the relative standard error exceeded 30%.

Prespecified sensitivity analyses excluded Panel 25, used the any-ED outcome, used three-level poverty, removed people with any emergency-room surgical/procedural events, excluded people with cancer, expanded exposure to all observed year-1 opioid prescriptions, separately added three alternative functional-limitation measures, categorized age, capped the top 1% of weights, and compared unadjusted and minimally adjusted models. Full specifications are provided in the Appendices.

After independent review, two explicitly exploratory analyses were added without changing the primary model. Nested count models sequentially added structural sociodemographic covariates, year-1 utilization, year-1 health status, and contemporaneous surgical/procedural setting and event-count variables. Setting-restricted full-model contrasts were estimated only when each exposure cell contained at least 50 participants and 30 ED-positive participants. Because participants could have qualifying events in multiple settings and no multiplicity adjustment was applied, these contrasts were not treated as confirmatory tests of setting heterogeneity.

## Results

Cohort and participant characteristics

The full pooled positive-weight survey universe contained 41,427 records. Out of 4,468 eligible adults with a surgical/procedural event linked to a condition, 4,454 (99.69%) had complete main-model data. The complete-case analysis excluded 14 of 4,468 participants (0.31%). All 7,772 surgical-event-condition links matched a condition public-use record, and all eligible participants matched the HC-036 variance file. The full survey design contained 117 strata and 400 stratum-primary sampling unit combinations, giving 283 design degrees of freedom. The analytic domain retained 261 design degrees of freedom.

Among complete cases, 816 of 4,454 participants (18.3%) had at least one observed condition-linked opioid prescription, and 3,638 of 4,454 (81.7%) did not. The corresponding average-panel weighted populations were approximately 5.77 million and 27.59 million. There were 194 of 816 ED-positive participants (23.8%) in the exposed group and 845 of 3,638 (23.2%) in the comparator group.

The weighted mean age was 57.23 years (N=4,454). Overall, 2,580 of 4,454 participants were women (survey-weighted: 56.9%), 1,305 of 4,454 were below 200% FPL (survey-weighted: 24.6%), and 2,776 of 4,454 had any private insurance (survey-weighted: 68.1%) (Table 1). Compared with the comparator group, adults with a condition-linked opioid prescription were younger on average (53.01 versus 58.12 years) and had higher year-1 mean ED visits (0.69 versus 0.47), inpatient discharges (0.61 versus 0.33), outpatient visits (3.82 versus 2.24), and office-based visits (18.44 versus 14.97). Walking limitation was more frequent in the exposed group (survey-weighted, 35.2% versus 23.9%; 1,321 of 4,454 participants reported a walking limitation overall). An inpatient setting was also more common as the highest-acuity setting among exposed participants (1,090 of 4,454 overall; survey-weighted, 23.2%), whereas an office-based setting was more common among the comparator group (1,929 of 4,454 overall; survey-weighted, 44.7%). These imbalances supported retaining the prespecified multivariable adjustment rather than treating the exposure groups as directly exchangeable.

Among the 4,468 eligible participants, exposure, outcome, demographic, poverty, insurance, year-1 utilization, and surgical/procedural-event variables had no missing values. Missingness occurred only in the main health-status covariates: physical health (five participants), mental health (three), walking limitation (seven), and cancer (one). These overlapped across participants, resulting in 14 exclusions and 4,454 complete cases. Each variable had at most 0.16% missingness, and the largest exposure-group difference was 0.26 percentage points (see Appendices).

Unadjusted year-2 emergency department outcomes

The unadjusted survey-weighted mean annual ED count was 0.372 (95% CI: 0.284-0.459) among adults with an observed condition-linked opioid prescription and 0.337 (95% CI: 0.303-0.372) among those without one (Table 2). The corresponding unadjusted proportions with any ED use were 194 of 816 (survey-weighted, 22.5%; 95% CI, 18.9%-26.1%) among adults with an observed condition-linked opioid prescription and 845 of 3,638 (survey-weighted, 21.5%; 95% CI, 19.7%-23.3%) among those without one.

**Table 2:** Unadjusted year-2 annual all-cause emergency department outcomes Estimates use the complete-case survey domain. For the "Any ED use" rows, N is the exact unweighted number of participants with at least one year-2 ED visit out of the unweighted n shown in the same row, and the percentage with its 95% CI is the survey-weighted proportion; because of complex survey weighting, N divided by the unweighted n will not equal the weighted percentage. Rows reporting mean ED visits give the survey-weighted mean with its 95% CI for the unweighted n shown. Weighted populations represent the average across four pooled two-year panel periods. "Linked opioid" denotes an observed year-1 opioid prescribed-medicine event linked to a condition also linked to a qualifying surgical/procedural event; "any" and "no" denote the presence or absence of at least one such event CI, confidence interval; ED, emergency department; FPL, federal poverty level

Exposure	Poverty group	Outcome	Estimate (95% CI)	Unweighted, n	Weighted population, millions
No observed linked opioid	All	Mean ED visits	0.337 (0.303-0.372)	3,638	27.590
Any observed linked opioid	All	Mean ED visits	0.372 (0.284-0.459)	816	5.767
No observed linked opioid	All	Any ED use	845 (21.5%; 19.7%-23.3%)	3,638	27.590
Any observed linked opioid	All	Any ED use	194 (22.5%; 18.9%-26.1%)	816	5.767
No observed linked opioid	At or above 200% FPL	Mean ED visits	0.276 (0.239-0.313)	2,583	20.935
Any observed linked opioid	At or above 200% FPL	Mean ED visits	0.279 (0.213-0.344)	566	4.210
No observed linked opioid	Below 200% FPL	Mean ED visits	0.530 (0.451-0.610)	1,055	6.654
Any observed linked opioid	Below 200% FPL	Mean ED visits	0.623 (0.358-0.887)	250	1.557

Unadjusted ED counts were higher in the below 200% FPL group than in the at or above 200% FPL group for both exposure categories. Among people below 200% FPL, mean counts were 0.623 (95% CI: 0.358-0.887) with and 0.530 (95% CI: 0.451-0.610) without a linked opioid prescription. Among people at or above 200% FPL, the corresponding means were 0.279 (95% CI: 0.213-0.344) and 0.276 (95% CI: 0.239-0.313).

Primary adjusted association

After standardization over the complete-case population, adjusted mean annual ED visits were 0.336 (95% CI: 0.275-0.412) under the any-linked-opioid exposure and 0.345 (95% CI: 0.311-0.382) under the no-observed-linked-opioid exposure (Table 3). The adjusted standardized mean ratio was 0.975 (95% CI, 0.784-1.211; p=0.8165). On the absolute scale, the adjusted mean difference was -0.009 visits per year (95% CI, -0.082 to 0.065; p=0.8150).

**Table 3:** Adjusted standardized year-2 emergency department outcomes Results are survey-standardized over the complete-case population (N=4,454); poverty-specific contrasts are standardized within poverty domains. Count models were adjusted for age, sex, race/ethnicity, region, poverty, insurance, year-1 ED, inpatient, outpatient, office-based utilization, physical and mental health, walking limitation, cancer, four surgical-setting indicators, surgical-event burden, and panel. The any-ED model used the same covariates. Probabilities, the risk difference, and the probability ratio are model-standardized over the complete-case population (N=4,454) and are not observed frequencies; the corresponding exact observed counts were 194 of 816 participants with and 845 of 3,638 without an observed condition-linked opioid prescription (Table 2). "Linked opioid" denotes an observed year-1 opioid prescribed-medicine event linked to a condition also linked to a qualifying surgical/procedural event; "any" and "no" denote the presence or absence of at least one such event CI, confidence interval; ED, emergency department; FPL, federal poverty level

Estimand	No observed linked opioid	Any observed linked opioid	Contrast (95% CI)	P-value
Mean annual ED visits	0.345 (0.311-0.382)	0.336 (0.275-0.412)	Ratio: 0.975 (0.784-1.211)	0.8165
Mean annual ED visits	0.345 (0.311-0.382)	0.336 (0.275-0.412)	Difference: -0.009 (-0.082 to 0.065)	0.8150
Probability of any ED use, standardized (N=4,454)	22.0% (20.2%-23.9%)	20.1% (16.8%-23.3%)	Probability ratio: 0.912 (0.760-1.094)	0.3195
Probability of any ED use, standardized (N=4,454)	22.0% (20.2%-23.9%)	20.1% (16.8%-23.3%)	Risk difference: -1.9 percentage points (-5.7 to 1.8)	0.3073
Mean visits, at or above 200% FPL	-	-	Ratio: 0.931 (0.721-1.202)	0.5819
Mean visits, below 200% FPL	-	-	Ratio: 1.036 (0.743-1.444)	0.8358
Poverty interaction effect scale	-	-	Ratio of count ratios: 1.112 (0.751-1.649)	0.5943
Exposure-by-poverty interaction	-	-	F=0.284	0.5943

The relative and absolute CIs included their respective null values and were compatible with associations in either direction. These estimates therefore did not establish equivalence or the absence of an association.

In the reviewer-requested nested specifications, the count ratio was 1.124 (95% CI: 0.878-1.439) after structural sociodemographic adjustment, 0.926 (95% CI: 0.741-1.156) after adding year-1 utilization, 0.964 (95% CI: 0.767-1.211) after adding year-1 health status, and 0.975 (95% CI: 0.784-1.211) in the full model. This movement across the null indicates specification dependence rather than a stable directional association (see Appendices).

Poverty interaction and any emergency department use

The exposure-by-poverty interaction did not provide evidence that the adjusted count association differed between poverty groups (F=0.284 and p=0.5943). The standardized any-versus-none mean ratio was 0.931 (95% CI: 0.721-1.202) among people at or above 200% FPL and 1.036 (95% CI: 0.743-1.444) among people below 200% FPL (Figure 2). The corresponding mean differences were -0.019 visits (95% CI: -0.087 to 0.049) and 0.019 visits (95% CI: -0.167 to 0.206). The interaction ratio of count ratios was 1.112 (95% CI: 0.751-1.649). Because the direct interaction did not support heterogeneity, these poverty-specific estimates are descriptive secondary contrasts and do not establish socioeconomic equity or inequity.

**Figure 2:**
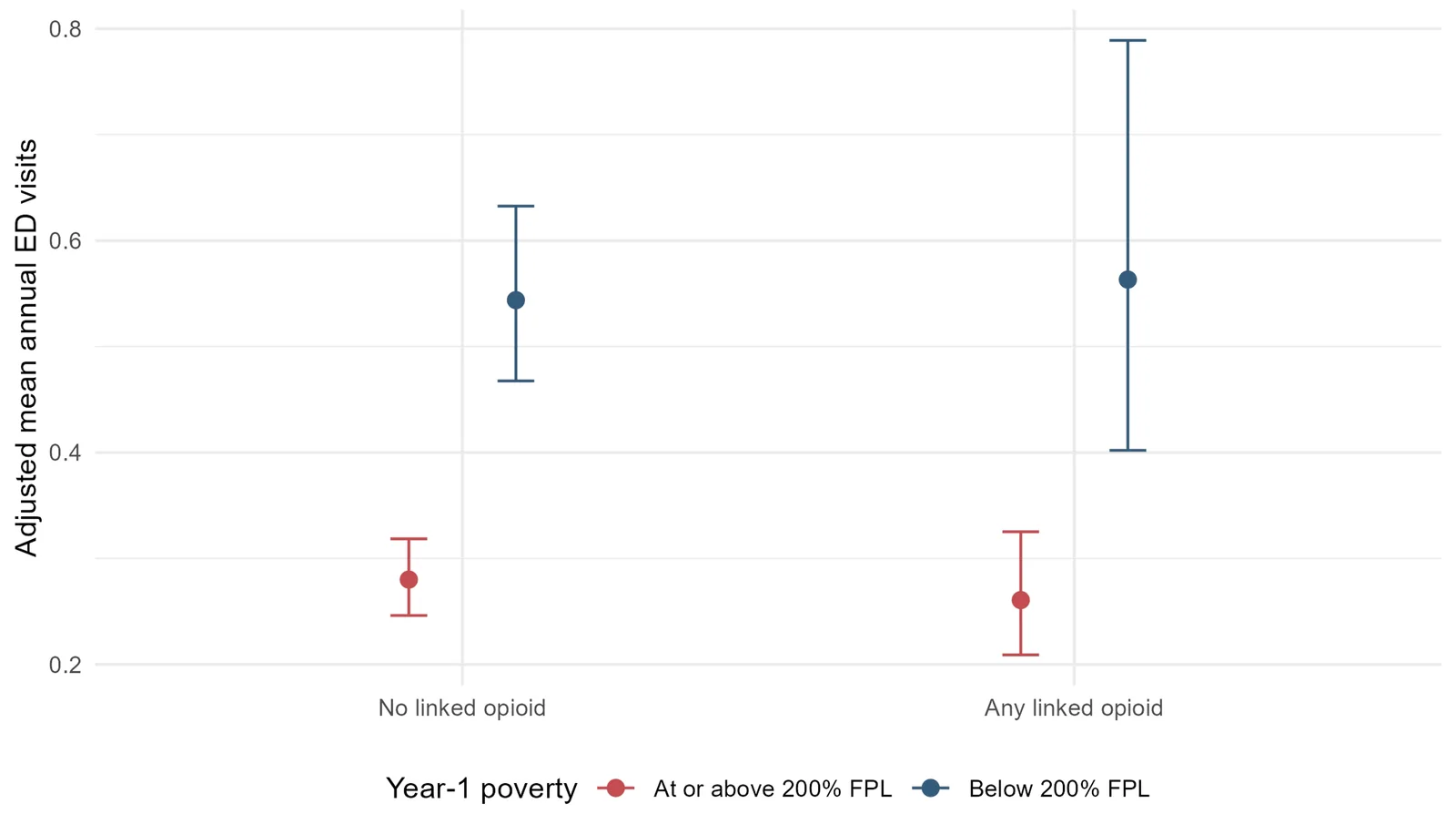
Adjusted mean year-2 annual all-cause emergency department visits by condition-linked opioid prescribed-medicine event and poverty group Points are survey-standardized adjusted means from the interaction model, and bars are 95% confidence intervals. Estimates are associational and come from a design-based quasi-Poisson model. Poverty groups are below 200% and at or above 200% of the federal poverty level (FPL). The direct exposure-by-poverty interaction was F=0.284, p=0.5943 ED: emergency department

For the secondary binary outcome, the adjusted probability of any year-2 ED use was 20.1% (95% CI: 16.8%-23.3%) with and 22.0% (95% CI: 20.2%-23.9%) without an observed condition-linked opioid prescription; these are model-standardized probabilities over the complete-case population (N=4,454) rather than observed frequencies, and the corresponding observed counts were 194 of 816 and 845 of 3,638 (Table 2). The adjusted risk difference was -1.9 percentage points (95% CI, -5.7 to 1.8; p=0.3073), and the adjusted probability ratio was 0.912 (95% CI, 0.760-1.094; p=0.3195).

"Any linked opioid" indicates one or more observed year-1 opioid prescribed-medicine events linked to the same condition as a surgical/procedural event. "No linked opioid" indicates none. The plotted exposure is not a temporally confirmed postoperative regimen.

Diagnostics and sensitivity analyses

All primary and secondary models converged with finite coefficients. The main count model had 32 parameters, 32.47 ED-positive participants per parameter, and a residual design degrees-of-freedom value of 261. The smallest modeled categorical level had 210 participants and 44 ED-positive participants. The quasi-Poisson dispersion estimate was 1.55, the weighted zero proportion was 78.3%, and the model-matrix condition number was 23.25. These results passed the prespecified diagnostic rules. Observed and predicted means agreed overall and within exposure groups; all 10 weighted prediction-group residual CIs included zero, and calibration-intercept and slope checks were approximately zero and one, respectively (see Appendices).

All prespecified sensitivity models converged. Count-ratio estimates remained null-spanning after excluding Panel 25 (1.002, 95% CI: 0.793-1.267), excluding people with emergency-room surgical/procedural events (0.987, 95% CI: 0.783-1.244), excluding people with cancer (0.915, 95% CI: 0.708-1.182), broadening the exposure to all observed year-1 opioid prescriptions (0.995, 95% CI: 0.833-1.188), adding alternative limitation variables (range: 0.969-0.980), categorizing age (0.964, 95% CI: 0.774-1.201), or capping the top 1% of sampling weights (0.978, 95% CI: 0.788-1.212). The unadjusted ratio was 1.101 (95% CI: 0.852-1.423), and the minimally adjusted ratio was 1.146 (95% CI: 0.893-1.469). The three-level poverty interaction also did not provide clear evidence of heterogeneity (p=0.2536). Full sensitivity estimates appear in the Appendices.

Exploratory setting-restricted ratios were 1.155 (95% CI: 0.828-1.610) for inpatient events, 0.675 (95% CI: 0.513-0.889) for outpatient events, and 1.034 (95% CI: 0.777-1.376) for office-based events. The emergency-room contrast was withheld because the exposed cell had only 22 ED-positive participants. Three of the four setting restrictions were therefore estimable, and one of those three excluded the null. Participants could contribute to more than one setting, no direct exposure-by-setting interaction was tested, and no multiplicity correction was applied across the family; these estimates therefore do not establish setting heterogeneity (see Appendices).

## Discussion

Principal findings

Using four longitudinal panels from MEPS, a survey of the US civilian noninstitutionalized population, we detected no clear adjusted association between an observed year-1 opioid prescribed-medicine event linked to the same condition as a surgical/procedural event and year-2 annual all-cause ED utilization in the eligible cohort. The analysis does not evaluate postoperative opioid prescribing because the procedure-medication sequence was not established. Standardized adjusted mean counts were close: a ratio of 0.975 and a difference of -0.009 visits per year. The secondary any-ED analysis pointed the same way. The point estimate, however, conceals the width around it. The primary CI spans a 21.6% lower to a 21.1% higher mean count on the ratio scale, a range wide enough to contain associations that would matter in either direction. These intervals leave clinically meaningful associations unresolved and do not support the equivalence or absence of an association.

The nested models showed that the estimate changed from above one after structural adjustment to below one after year-1 utilization was added and then moved toward one with health and event-level adjustment. This pattern reinforces the importance of confounding by indication and contemporaneous adjustment; it does not identify which specification is causal. The outpatient-restricted estimate was lower than one, but the overlapping-setting definition, sparse emergency-room support, absence of a direct heterogeneity test, and multiple exploratory comparisons preclude a setting-specific clinical conclusion.

The prespecified poverty interaction likewise did not provide evidence of heterogeneity. Point estimates fell on opposite sides of the null in the two poverty groups, but both CIs were wide, and the direct interaction p-value was 0.5943. Interpreting separate subgroup estimates as different would therefore be inappropriate. The result is better understood as an inconclusive test of effect-measure heterogeneity than as evidence that socioeconomic differences are absent.

Comparison with prior literature

Our findings address a different estimate from earlier MEPS studies. Okeke et al. reported an association between opioid use and ED visits among adults with spine-related disorders using annual data and a zero-inflated count model [6]. Turk et al. examined opioid prescriptions and hospital-based services over two-year panels among adults with disability or inflammatory conditions [7]. The present analysis narrows exposure attribution to a condition shared with a surgical/procedural event, orders exposure and outcomes across years, and reports standardized absolute and relative contrasts. These differences in population, linkage, temporal ordering, covariates, and estimands limit direct numerical comparison.

The longitudinal ordering follows the general approach of MEPS panel studies that placed health or opioid measures in one year and outcomes in the next [8,9]. Adjustment changed the exposure contrast from an unadjusted ratio above one to an adjusted ratio near one, consistent with substantial year-1 differences in utilization, health status, and procedural setting. This shift does not prove that confounding was eliminated. It instead highlights how strongly indication, care intensity, and health status may shape both an observed prescribed-medicine event and later acute-care use.

Hernandez et al. recently used longitudinal MEPS data, matching, and difference in differences to estimate financial hardship after surgical procedures and variation by payer and income [13]. That work provides a contemporary surgery/equity design benchmark, but it addresses financial outcomes rather than condition-linked opioid prescriptions and subsequent ED use. The present study's incremental contribution is, therefore, the condition-event-prescription linkage, year-separated ED outcome, and direct poverty interaction, not a claim to be the first longitudinal MEPS study of surgical outcomes.

The study also differs from exact postoperative claim analyses. Brummett et al. used a procedure index, baseline opioid status, perioperative dispensing, and a 90- to 180-day outcome window to study persistent use [14]. Sutherland et al. used procedure-indexed claims to estimate postsurgical dispensing trends [15]. Howard et al. evaluated a localized prescribing guideline using a comparison group and difference-in-differences design [16]. Those data support timing and policy questions that public-use MEPS cannot answer. Conversely, MEPS contributes self-reported health, function, poverty, insurance, and broad utilization measures for the civilian noninstitutionalized population.

Socioeconomic interpretation

Earlier research has documented social patterning in opioid prescribing [10-12]. Those differences can reflect variation in clinical need, access, clinician decision-making, insurance, and structural inequity, and our design separates none of them.

One distinction governs how our result should be read. We did not test whether the prevalence of observed opioid medicine events differed by poverty. We tested whether the adjusted association between a condition-linked opioid prescribed-medicine event and subsequent ED use differed across two poverty groups. A nonsignificant interaction on the second question is silent on the first. It does not negate documented disparities, and it should not be described as evidence of equity. The wide poverty-specific CIs leave little power to detect modest heterogeneity in any case, and a binary family-income threshold remains a thin proxy for socioeconomic position: it cannot represent wealth, neighborhood conditions, care access, or health literacy.

Strengths

The condition-event-medication algorithm required an opioid prescribed-medicine event and a surgical/procedural event to share a condition within year 1. This restriction improves specificity relative to classifying every opioid medicine event in a person with a procedure as procedure-related. It does not confirm the clinical indication, temporal sequence, or membership in the same treatment episode. The two-year panel design establishes year-level ordering between the exposure year and the subsequent ED outcome year.

Design handling was similarly conservative. The analysis retained the full pooled survey universe for design construction and used domain analysis, common HC-036 strata and sampling units, and longitudinal weights, so variance estimates reflect the released design rather than a subset rebuilt after exclusions.

Reporting choices favored interpretability over emphasis. Standardized means and absolute differences accompany every relative estimate, so a reader can see the size of the contrast on the scale of visits rather than only as a ratio. The poverty question was answered with a direct interaction test rather than by comparing two separately fitted subgroup models, and sparse model levels were collapsed under thresholds set in advance. The protocol, deviation log, stable result keys, diagnostics, sensitivity analyses, and two-run hash comparison make the full chain from public-use file to reported estimate auditable.

Limitations

First, and most consequential for interpretation, is temporal ambiguity within year 1. The condition linkage does not establish whether medication acquisition preceded or followed the surgical/procedural event, so this analysis cannot estimate a postoperative association or a short-term postoperative revisit outcome. Although MEPS includes medication strength, quantity, days supplied, and coarse timing fields, the binary exposure used here did not characterize dose, duration, morphine milligram equivalents, or a procedure-specific regimen. These records do not establish actual consumption, adherence, or a complete analgesic strategy [17].

Second, residual confounding by indication remains likely. Adults with an observed linked opioid prescribed-medicine event differed in year-1 utilization, functional limitation, and procedural setting. Pain severity, prior opioid exposure, procedure complexity, acute complications, clinician judgment, and other clinical factors were incompletely measured. Year-1 utilization and procedural variables may both reflect clinical severity and occur after medication acquisition. Adjustment for a downstream variable could remove part of an association of interest; conditioning on a common consequence of exposure and unmeasured illness could introduce collider bias. These are possible sources of bias, not mechanisms demonstrated by this analysis. The nested estimates' movement across the null shows that interpretation depends on the adjustment specification and does not identify a causal model.

Third, the outcome was annual all-cause ED use. It is a broad utilization signal, not a postoperative revisit measure, and cannot be attributed to the surgery-associated condition, pain management, medication adverse events, or any particular mechanism. Household-reported healthcare use may be underreported. Validation work has identified the incomplete reporting of ED use in MEPS [22]. Nondifferential outcome underreporting could reduce precision and attenuate some contrasts, whereas differential reporting could bias estimates in either direction.

Fourth, prescribed-medicine events reflect household-reported acquisition, with pharmacy-derived information supplementing medication characteristics; they do not verify ingestion or adherence. The no-observed-linked-opioid comparator may include unreported acquisitions, medicines provided during inpatient care, nonprescription treatments, or opioid events linked to a different condition. Exposure misclassification could attenuate or otherwise distort the association.

Fifth, the target population was limited to adults represented by four complete two-year panels who had a broad surgery/procedure indicator and a linkable condition. It excludes institutionalized people and does not represent every calendar year. Weighted longitudinal estimates incorporate MEPS adjustments for panel participation, but selection into complete panels can still limit generalizability. The pooled periods also include the coronavirus disease 2019 pandemic. Excluding the panel with 2020 as the exposure year did not materially change the estimate, but the sensitivity analysis cannot remove every pandemic-related change in care.

The broad procedural indicators encompass clinically heterogeneous encounters. In Table 1, an inpatient encounter was the highest-acuity setting for 44.1% of the exposed group versus 18.9% of the comparator group, whereas the corresponding office-based proportions were 18.5% and 50.1%. These are survey-weighted descriptive categories; adjustment for setting and event burden does not make the groups clinically equivalent. The analysis also omits the 2019-2020 Panel 24 period and cannot establish that the findings generalize to that omitted period.

Finally, the primary estimate was imprecise. The CI included associations in both directions that could matter clinically or operationally, and the poverty-specific intervals were wider. The lack of statistical evidence should not be interpreted as a confirmation of a clinically negligible association.

Only 816 complete-case participants were classified as exposed, including 250 below 200% FPL and 566 at or above that threshold. The wide subgroup intervals limit the ability to distinguish modest effect modification from sampling uncertainty.

Implications and future research

These findings should not be used to recommend for or against opioid prescribing for an individual patient. They instead clarify what can be learned from condition-linked longitudinal survey data and where stronger data are required. For clinicians, the estimates do not compare analgesic regimens; for health-system leaders, they do not evaluate a specific opioid-sparing protocol; and for policymakers, they show that public-use MEPS alone cannot measure equitable protocol adoption. A definitive perioperative study would combine exact procedure dates, procedure type and complexity, baseline opioid history, prescription dates, dose and duration, multimodal treatment components, and dated cause-specific acute-care encounters. Claims or electronic health record data could support those measurements, while linkage to socioeconomic information could preserve the equity question.

Future work should prespecify clinically meaningful time windows and assess whether associations vary by procedure, baseline opioid exposure, pain severity, insurance, and more granular socioeconomic measures. Where a specific guideline or institutional program is evaluated, a concurrent comparison group and a clearly dated intervention would strengthen causal interpretation. National survey analyses remain useful for describing population patterns, but their claims should remain aligned with their year-level measurement.

## Conclusions

Among eligible adults in four pooled longitudinal MEPS panels, no clear adjusted association was detected between an observed year-1 opioid prescribed-medicine event linked to the same condition as a year-1 surgical/procedural event and year-2 annual all-cause ED use. The adjusted mean count ratio was 0.975 (95% CI: 0.784-1.211), and the prespecified exposure-by-poverty interaction did not provide evidence of heterogeneity (ratio of count ratios, 1.112; 95% CI, 0.751-1.649).

The confidence intervals remain compatible with clinically meaningful associations and do not establish equivalence, the absence of an association, or socioeconomic equity. Because medication acquisition was not temporally ordered relative to the procedure, these results do not establish the effects or safety of postoperative opioid prescribing or the effectiveness of an analgesic regimen.

## Appendices

Supplementary material

Table 4 shows the exact public-use file and panel-year crosswalk.

**Table 4:** Public-use files and panel-year crosswalk The common variance-estimation fields STRA9623 and PSU9623 came from HC-036. Panel-file VARSTR and VARPSU fields were not combined with the HC-036 fields. The analysis used the four panels listed in this table. Panel 24 (2019-2020), available as the two-year HC-225 file, was not included. For Panel 22, HC-036 contained some legacy eight-character 2017 person identifiers and harmonized 2018 representations. The extraction program prepended 22 to the legacy identifiers only after selecting Panel 22, verified that duplicate transformed keys had identical variance fields, and then deduplicated them. This yielded complete HC-036 linkage for all 41,427 positive-weight universe records and all 4,468 eligible records

Panel	Exposure year	Outcome year	Longitudinal file	Year-1 prescribed medicines	Year-1 event files	Event-condition link	Conditions
22	2017	2018	HC-210	HC-197A	HC-197D/E/F/G	HC-197I	HC-199
23	2018	2019	HC-217	HC-206A	HC-206D/E/F/G	HC-206I	HC-207
25	2020	2021	HC-234	HC-220A	HC-220D/E/F/G	HC-220I	HC-222
26	2021	2022	HC-244	HC-229A	HC-229D/E/F/G	HC-229I	HC-231

Table 5 shows the variable definitions and coding.

**Table 5:** Main variable definitions and coding ED: emergency department

Construct	Source/derivation	Main-analysis coding
Condition-linked opioid prescribed-medicine event	Multum class 60 or 191 plus EVENTYPE=8 linkage to a surgical-event condition	Any versus no observed qualifying record
Year-2 ED count	ERTOTY2	Annual all-cause count
Any year-2 ED use	ERTOTY2>0	Binary
Poverty	POVCATY1	Below 200% versus at or above 200% federal poverty level
Age	AGEY1X	Natural spline, three degrees of freedom
Sex	Released category	Male/female
Race/ethnicity	RACETHX	White, Hispanic, Black, and Asian/other/multiple for adjustment; five released groups descriptively
Region	REGIONY1	Northeast, Midwest, South, West
Insurance	INSCOVY1	Any private versus no private for adjustment; three released groups descriptively
Baseline utilization	ERTOTY1, IPDISY1, OPTOTVY1, OBTOTVY1	log(1+x)
Physical health	RTHLTH1	Excellent/very good, good, fair/poor
Mental health	MNHLTH1	Excellent/very good, good, fair/poor
Walking limitation	WLKLIM1	Yes/no
Cancer	CANCERY1	Yes/no
Surgical settings	Derived from event files	Four concurrent indicators
Surgical-event burden	Count of unique qualifying events	log(1+x)
Panel	Panel number	Four fixed categories

Supplementary model specification and coding

The unit of analysis was one person within a selected panel. Qualifying events were deduplicated by DUPERSID and EVNTIDX and condition pairs by DUPERSID and CONDIDX. A person with multiple qualifying events or medication acquisitions contributed once; any qualifying opioid event set the exposure indicator to one. No procedure-specific diagnosis-code list was required: eligibility was based on ANYOPER=1 or SURGPROC=1 plus a valid condition link. Surgical/procedural burden counted unique qualifying events, and the four setting indicators were concurrent rather than mutually exclusive.

The primary count model included the binary exposure; a three-degree-of-freedom natural spline for AGEY1X; sex, race/ethnicity, region, poverty, insurance, physical and mental health, walking limitation, cancer, and panel; log(1+x) transformations of ERTOTY1, IPDISY1, OPTOTVY1, OBTOTVY1, and the unique event count; and four surgical/procedural-setting indicators. It used a quasi-Poisson family and log link without a person-time offset because the outcome was an annual count for the defined year-2 period. The interaction model added the exposure-by-below-200%-FPL product term with both main effects retained. The any-ED model used the same main covariates with a quasibinomial family and logit link.

Reference categories were no observed linked opioid event, male sex (SEX=1), non-Hispanic White (RACETHX=2), Northeast (REGIONY1=1), at or above 200% FPL (POVCATY1=4-5), any private insurance (INSCOVY1=1), excellent/very good physical and mental health (codes 1-2), no walking limitation or cancer (code 2), the absence of each setting indicator, and Panel 22. Health code 3 was good, and codes 4-5 were fair/poor. Walking limitation and cancer used 1=yes and 2=no; invalid values were missing. Race/ethnicity categories 4-5 were combined for adjustment, and insurance categories 2-3 were combined as no private coverage.

The prespecified collapsing rules required at least 50 observations and 30 ED-positive participants per categorical adjustment level. The separate Asian category had six ED-positive participants, and the uninsured category had 17, motivating the combinations above. The complete-case policy was prespecified; 14 of 4,468 eligible participants were excluded, and Table 6 reports variable-specific and exposure-group missingness. No additional imputation or missing-data sensitivity analysis was performed. Survey design, weights, standardization, variance estimation, and diagnostic checks are specified in the Materials & Methods section and Tables 7-10.

**Table 6:** Main-model missingness overall and by exposure Fourteen participants were excluded due to the union of missing main covariates, for a complete-case loss of 0.31%. "Linked opioid" denotes an observed year-1 opioid prescribed-medicine event linked to a condition also linked to a qualifying surgical/procedural event; "any" and "no" denote the presence or absence of at least one such event

Variable	Missing, N	Overall, %	No linked opioid, %	Any linked opioid, %	Absolute difference, percentage points
Physical health	5	0.112	0.110	0.122	0.012
Mental health	3	0.067	0.055	0.122	0.067
Walking limitation	7	0.157	0.110	0.366	0.256
Cancer	1	0.022	0.027	0.000	0.027
All other main variables	0	0.000	0.000	0.000	0.000

**Table 7:** Primary exposure coefficients The M3 odds ratio is supplied only as a model coefficient; the main manuscript prioritizes standardized probabilities, the risk difference, and the probability ratio. "Linked opioid" denotes an observed year-1 opioid prescribed-medicine event linked to a condition also linked to a qualifying surgical/procedural event; "any" and "no" denote the presence or absence of at least one such event ED, emergency department; FPL, federal poverty level; CI, confidence interval

Model	Exposure-related term	Coefficient	Standard error	Exponentiated estimate (95% CI)	P-value
M1 count	Any linked opioid	-0.0257	0.1104	0.975 (0.784-1.211)	0.8165
M2 count interaction	Any linked opioid at ≥200% FPL	-0.0716	0.1298	0.931 (0.721-1.202)	0.5819
M2 count interaction	Any linked opioid×below 200% FPL	0.1066	0.1999	1.112 (0.751-1.649)	0.5943
M3 any ED	Any linked opioid	-0.1344	0.1339	Odds ratio: 0.874 (0.672-1.138)	0.3165

**Table 8:** Observed-versus-predicted calibration for the primary count model Prediction groups were formed from weighted quantiles of fitted values. Every observed-minus-predicted 95% CI included zero. The calibration intercept was approximately 0 and the slope approximately 1; these are in-sample descriptive checks, not external validation. "Linked opioid" denotes an observed year-1 opioid prescribed-medicine event linked to a condition also linked to a qualifying surgical/procedural event; "any" and "no" denote the presence or absence of at least one such event CI: confidence interval

Group	Observed mean	Predicted mean	Observed minus predicted (95% CI)	N
Overall	0.343	0.343	0.000 (-0.028 to 0.028)	4,454
No linked opioid	0.337	0.337	0.000 (-0.032 to 0.032)	3,638
Any linked opioid	0.372	0.372	0.000 (-0.071 to 0.071)	816
Prediction group 1	0.070	0.104	-0.033 (-0.070 to 0.003)	341
Prediction group 2	0.132	0.129	0.003 (-0.056 to 0.061)	414
Prediction group 3	0.183	0.151	0.032 (-0.077 to 0.141)	399
Prediction group 4	0.160	0.174	-0.015 (-0.063 to 0.034)	427
Prediction group 5	0.179	0.208	-0.029 (-0.086 to 0.029)	452
Prediction group 6	0.311	0.249	0.063 (-0.016 to 0.142)	427
Prediction group 7	0.303	0.307	-0.004 (-0.088 to 0.080)	441
Prediction group 8	0.350	0.386	-0.037 (-0.106 to 0.032)	467
Prediction group 9	0.578	0.536	0.042 (-0.063 to 0.146)	516
Prediction group 10	1.169	1.190	-0.021 (-0.180 to 0.137)	570

**Table 9:** Sensitivity-analysis specifications ED: emergency department

ID	Specification	Purpose
S01	Exclude Panel 25	Remove the 2020 exposure year
S02	Any year-2 ED use	Test outcome functional form
S03	Three-level poverty interaction	Test socioeconomic grouping
S04	Exclude anyone with an emergency-room surgical event	Remove emergency-room-identified surgery
S05	Exclude people with cancer	Reduce cancer-related heterogeneity
S06	Classify all observed year-1 opioid prescribed-medicine events	Test condition-linkage sensitivity
S07A-C	Add activity, instrumental-activity, and activity-of-daily-living limitations separately	Test functional-status specification
S08	Categorize age	Test age functional form
S09	Cap the top 1% of weights	Assess influential weights
S10A-B	Unadjusted and minimally adjusted models	Display adjustment impact

**Table 10:** Count-model sensitivity results The three-level poverty interaction Wald test was F=1.381, p=0.2536. Every sensitivity model converged, and every reported interval included the null CI: confidence interval

Analysis	N	Standardized mean ratio (95% CI)	Standardized mean difference (95% CI)	Ratio p-value
Primary fully adjusted	4,454	0.975 (0.784-1.211)	-0.009 (-0.082 to 0.065)	0.8165
Exclude Panel 25	3,809	1.002 (0.793-1.267)	0.001 (-0.084 to 0.085)	0.9854
Exclude emergency-room surgical events	4,141	0.987 (0.783-1.244)	-0.004 (-0.080 to 0.071)	0.9097
Exclude cancer	3,228	0.915 (0.708-1.182)	-0.029 (-0.111 to 0.052)	0.4939
All year-1 opioid prescriptions	4,454	0.995 (0.833-1.188)	-0.002 (-0.063 to 0.059)	0.9514
Add activity limitation	4,446	0.969 (0.777-1.209)	-0.011 (-0.085 to 0.064)	0.7785
Add instrumental-activity limitation	4,452	0.974 (0.783-1.211)	-0.009 (-0.083 to 0.065)	0.8102
Add activity-of-daily-living limitation	4,450	0.980 (0.786-1.221)	-0.007 (-0.081 to 0.067)	0.8547
Age categories	4,454	0.964 (0.774-1.201)	-0.012 (-0.086 to 0.061)	0.7432
Cap top 1% of weights	4,454	0.978 (0.788-1.212)	-0.008 (-0.081 to 0.065)	0.8360
Unadjusted	4,454	1.101 (0.852-1.423)	0.034 (-0.060 to 0.128)	0.4616
Minimally adjusted	4,454	1.146 (0.893-1.469)	0.049 (-0.045 to 0.142)	0.2830

Supplementary results

Cohort Construction

Table 11 shows the panel-specific cohort flow.

**Table 11:** Panel-specific cohort flow All 7,772 surgical-event-condition links matched a condition-file record

Panel	Longitudinal records	Complete-panel adults	Adults with surgical event	Adults with linked surgical condition	Eligible adults	Complete cases
22	14,541	10,258	2,016	1,825	1,507	1,497
23	14,067	10,084	2,069	1,832	1,481	1,480
25	6,078	4,599	1,045	923	646	645
26	6,741	5,193	1,243	1,089	834	832
Total	41,427	30,134	6,373	5,669	4,468	4,454

The event-file and linked-condition counts precede intersection with the complete-panel adult longitudinal cohort. They should not be read as successive adult exclusions. Eligibility required that intersection, a positive longitudinal weight, age of at least 18 years, and ALL5RDS=1.

Model-Level and Diagnostic Checks

The smallest adjustment level after collapsing contained 210 participants and 44 ED-positive participants. The main count model used 32 parameters, giving 32.47 ED-positive participants per parameter. All M1-M3 models converged with finite coefficients. The quasi-Poisson dispersion estimate was 1.552, the weighted zero proportion was 0.783, and the model-matrix condition number was 23.25.

Additional analyses and supporting results are presented in Tables 12-14.

**Table 12:** Reviewer-requested nested adjustment analysis Year-1 utilization, health status, setting, and event count may be contemporaneous with exposure. The sequence is a specification analysis, not a causal mediation decomposition CI: confidence interval

Model	Added covariate block	Standardized mean ratio (95% CI)	Standardized mean difference (95% CI)
N1	Structural sociodemographic covariates	1.124 (0.878-1.439)	0.042 (-0.050 to 0.133)
N2	N1 plus year-1 utilization	0.926 (0.741-1.156)	-0.026 (-0.099 to 0.047)
N3	N2 plus year-1 health status	0.964 (0.767-1.211)	-0.013 (-0.089 to 0.064)
N4	N3 plus surgical/procedural setting and event count (primary full model)	0.975 (0.784-1.211)	-0.009 (-0.082 to 0.065)

**Table 13:** Setting-by-exposure support Survey-weighted populations use the pooled longitudinal weight LONGWT/4. Weighted shares are calculated within each setting. Participants may appear in more than one setting, so rows must not be summed across settings. The prespecified support rule required an unweighted N of at least 50 and at least 30 participants with any year-2 ED use in both exposure cells. "Linked opioid" denotes an observed year-1 opioid prescribed-medicine event linked to a condition also linked to a qualifying surgical/procedural event; "any" and "no" denote the presence or absence of at least one such event ED: emergency department

Setting	Exposure	Unweighted, N	Survey-weighted population, millions	Weighted share within setting	ED-positive, N	Support status
Inpatient	No linked opioid	726	5.21	67.2%	238	Supported
Inpatient	Any linked opioid	364	2.54	32.8%	97	Supported
Emergency room	No linked opioid	246	1.79	80.3%	92	Supported
Emergency room	Any linked opioid	67	0.44	19.7%	22	Withheld: ED-positive, N<30
Outpatient	No linked opioid	1,071	8.11	76.2%	260	Supported
Outpatient	Any linked opioid	355	2.54	23.8%	75	Supported
Office-based	No linked opioid	2,256	17.24	89.0%	460	Supported
Office-based	Any linked opioid	301	2.14	11.0%	80	Supported

**Table 14:** Reviewer-requested setting-restricted exploratory contrasts Participants may appear in more than one setting restriction. No direct exposure-by-setting interaction or multiplicity correction was applied, so these estimates do not establish heterogeneity ED, emergency department; CI, confidence interval

Setting restriction	N	Standardized mean ratio (95% CI)	Standardized mean difference (95% CI)	Status
Inpatient	1,090	1.155 (0.828-1.610)	0.070 (-0.100 to 0.239)	Estimated
Emergency room	313	-	-	Withheld: exposed cell had 22 ED-positive participants
Outpatient	1,426	0.675 (0.513-0.889)	-0.126 (-0.210 to -0.042)	Exploratory
Office-based	2,557	1.034 (0.777-1.376)	0.010 (-0.079 to 0.099)	Estimated

Ethics and Data Availability

MEPS public-use files are deidentified and publicly available from the Agency for Healthcare Research and Quality [17]. The analysis used only deidentified, publicly available secondary data and did not involve human subjects as defined under 45 CFR 46.102. Institutional review board review and informed consent were therefore not required. The input public-use files, file identifiers, and official download locations are documented in the supplement.

Code and materials availability statement: The two analysis programs, the frozen statistical protocol, the dated deviation log, the variable and therapeutic-class crosswalk, and all aggregate output artifacts are openly available at https://doi.org/10.5281/zenodo.21693654, archived from https://github.com/Tigerpoint/meps-condition-linked-opioid-ed. The repository includes a script that retrieves every Medical Expenditure Panel Survey public-use file used here from the Agency for Healthcare Research and Quality and verifies each against a recorded SHA-256 manifest. No person-level data are distributed; the temporary person-level modeling table is written outside the repository and deleted after each successful run. The pipeline is deterministic, and an independent end-to-end re-execution from the source public-use files reproduced every reported estimate and all archived aggregate artifacts.
